# Resistance Training Exercise Program for Intervention to Enhance Gait Function in Elderly Chronically Ill Patients: Multivariate Multiscale Entropy for Center of Pressure Signal Analysis

**DOI:** 10.1155/2014/471356

**Published:** 2014-09-10

**Authors:** Ming-Shu Chen, Bernard C. Jiang

**Affiliations:** ^1^Department of Industrial Engineering and Management, Yuan Ze University, Taoyuan, Chung-Li 32003, Taiwan; ^2^Department of Health Management Center, Far Eastern Memorial Hospital, No. 21, Sec. 2, Nanya S. Rd., Banciao District, New Taipei City 220, Taiwan; ^3^Department of Industrial Management, National Taiwan University of Science and Technology, No. 43, Sec. 4, Keelung Road, Da'an District, Taipei City 10607, Taiwan

## Abstract

Falls are unpredictable accidents, and the resulting injuries can be serious in the elderly, particularly those with chronic diseases. Regular exercise is recommended to prevent and treat hypertension and other chronic diseases by reducing clinical blood pressure. The “complexity index” (CI), based on multiscale entropy (MSE) algorithm, has been applied in recent studies to show a person's adaptability to intrinsic and external perturbations and widely used measure of postural sway or stability. The multivariate multiscale entropy (MMSE) was advanced algorithm used to calculate the complexity index (CI) values of the center of pressure (COP) data. In this study, we applied the MSE & MMSE to analyze gait function of 24 elderly, chronically ill patients (44% female; 56% male; mean age, 67.56 ± 10.70 years) with either cardiovascular disease, diabetes mellitus, or osteoporosis. After a 12-week training program, postural stability measurements showed significant improvements. Our results showed beneficial effects of resistance training, which can be used to improve postural stability in the elderly and indicated that MMSE algorithms to calculate CI of the COP data were superior to the multiscale entropy (MSE) algorithm to identify the sense of balance in the elderly.

## 1. Introduction

In an aging society, an increasing number of elderly adults are suffering from chronic diseases, such as hypertension, hyperlipidemia, heart disease, diabetes, and/or osteoporosis. Falls are unpredictable accidents, and the resulting injuries can be serious in the elderly, particularly those with chronic diseases.

Falling in the hospital is a patient safety issue and a serious medical concern for the elderly in general. According to reports by the Taiwan Joint Commission on Hospital Accreditation (2001), falls present a probable injurious event in hospitalized patients accompanied by the risk of marginal flesh wound (bruises) in less severe cases and fractures and internal bleeding in severe cases. This is particularly seen in those aged >65 years because accidental injuries are the second leading cause of death in this group. Although the incidence of falling is ever-increasing in an aging society, preventative measures are lacking in elderly communities. Mesa et al. evaluated 192 falling events involving 171 elderly patients of the 12,894 patients registered in their hospital between March 1996 and May 1998. They analyzed the circumstances of the fall and characteristics of those who suffered from more than one fall. They reported that the risk of a fall increased with age, with the majority occurring in those who were receiving rehabilitation therapy following a vascular cerebral accident [1]. Bischoff-Ferrari et al. reported that falls among the elderly occur more frequently because of advanced age and lead to substantial morbidity and mortality [2]. Honeycutt and Ramsey investigated factors related to falls in elderly men and the differences in characteristics between those who experienced a fall and those who did not [3]. Their results showed that in ambulatory elderly men, those who were underweight with slow gait time and poor balance had the highest risk of falling. In all the aforementioned reports, it is shown that increasing age was associated with a greater risk of falling. However, a further empirical analysis is required to determine methods to effectively decrease the occurrence of falls in the elderly community or hospital setting.

Exercise training and nutritional supplementation are commonly considered to enhance gait and balance function and health for elderly people [4]. The development of better methods to enhance balance for preventing falls in the elderly in a community or hospital setting has become a very important issue as well as a popular research topic. For example, Lai et al. demonstrated that static balance training is an effective method of increasing the balancing capacity of aged adults and reducing their fall risk [5]. Sparrow et al. concluded that exercise intervention improved patient strength and balance because group differences were statistically significant for knee flexion strength (*P* = 0.035) and single-leg stance time (*P* = 0.029). Moreover, resistance training exercise programs have been found to improve overall muscle strength and balance in middle-aged and older adults [6]; therefore, to evaluate the effectiveness of this type of intervention, future studies should be designed on the basis of more distal health- and disability-related outcomes in older adults. Regular exercise, such as aerobic training, reduces clinical blood pressure, and is thus recommended for the prevention and treatment of hypertension and other chronic diseases [7]. Hydraulic resistant exercise can be recommended for those with osteoporosis for improving bone density [8]. Martinez-Amat et al. implemented a new training program to evaluate the effect of a 12-week specific proprioceptive training program on postural stability, gait, balance, and fall prevention in adults >65 years of age. The training program improved postural balance of older adults in the mediolateral plane (ML) with eyes open (*P* < 0.05) and anteroposterior plane (AP) with eyes closed (*P* < 0.01). Further, significant improvements were observed in the Romberg quotient regarding surface (*P* < 0.05) and speed (*P* < 0.01) but not distance (*P* > 0.05). After proprioception training, gait (Tinetti) and balance (Berg) test scores improved by 14.66% and 11.47%, respectively [9].

Based on the conclusions of the aforementioned studies, exercise or strength training programs can enhance balance in elderly, but in elderly, chronically ill patients, it is unknown whether the same results can be achieved. Therefore, the first purpose of the present study was to evaluate whether regular exercise or resistance training can enhance gait function in elderly patients with chronic diseases, such as cardiovascular disease (CVD), diabetes mellitus (DM), or osteoarthritis (OA) and/or osteoporosis (OP).

Most commonly used methods to measure the sense of balance and gait are the single-leg stance time with open or closed eyes (measured in seconds), the timed up-and-go test (s), and the 30 s sit-to-stand test, among others. However, some recent studies regarding gait and balance performance used the patient's center of pressure (COP) as a factor to calculate the complexity index (CI) of multiscale entropy (MSE), including anterioposterior plane (AP), mediolateral plane (ML), and other balance/gait traditional indices to evaluate a patient's postural sway [10]. Recently published research about the multivariate multiscale entropy (MMSE) method has been validated on both illustrative benchmark signals and on a simultaneous analysis of the ML and AP components of postural sway dynamics from both young and elderly subjects [11].

Although MSE has gradually been applied in clinical gait function analysis tools, its variation is too large. The advanced MMSE algorithm improves the MSE deficiencies and has been demonstrated as an enabling tool for the complexity analysis of real-world multivariate data; however, the MMSE method's clinical application is mainly limited to analyzing the electroencephalogram (EEG) signal of critically ill patients to help distinguish whether they are in a coma or a quasi-brain-death (QBD) state. The advantages of MMSE have been exemplified for detection signatures caused by increased cognitive load and stress, highlighting its appeal in human-centered applications [12]. Ahmed et al. recorded the EEG data from the intensive care unit in Hua Shan Hospital, Shanghai, China, using a standardized 10–20 system. They analyzed 34 patients (16 female, 18 male) of ages ranging from 17 to 85 years old; 17 of the patients were in coma and 17 in the QBD state. The findings of their study support that the MMSE method can characterize states of brain consciousness from multichannel EEG recordings, with more conclusive results than the univariate MSE method [13].

Ahmed et al. published another article about the application of MMSE to coma and QBD EEG data analysis. They offered a detailed description of the MMSE algorithm, and once again they show that the MMSE method is more suitable in the clinical application of EEG data analysis than MSE method [14].

Although those studies have used MMSE-related indicators in clinical application, our study is novel in applying MMSE to exercise intervention for elderly, chronically ill patients to compare the difference in gait function. Therefore, this study also aims to verify whether the MMSE from the COP signal analysis can be a balance indicator applied to clinical gait analysis and can discriminate between other gait function indicators, particularly the traditional indices of MSE, balance, and gait.

## 2. Materials and Methods

A 12-week circuit hydraulic resistance training program was implemented to improve postural stability, gait, and balance and prevent falls prevention in adults >45 years of age. We separately selected groups of 10 patients with cardiovascular disease (CVD), diabetes mellitus (DM), or osteoarthritis (OA) and/or osteoporosis (OP) to join the exercise experimental groups. All patients signed Institutional Review Board (IRB) agreements before participation (IRB approval number: FEMH-IRB-101029-E, v. 02, Date: 20120429). Because of all chronically ill patients among the participants needed to complete the signed IRB agreements before participation and finished 12-week training. Thus, it is a challenge to invite volunteers to participate in this study.

### 2.1. Resistance Training Methods

**Figure 1:**
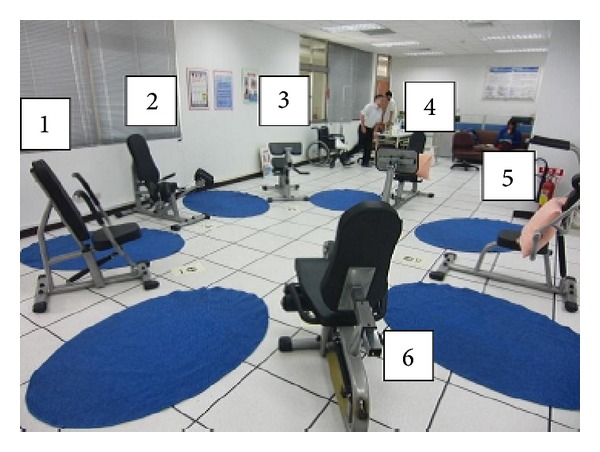
The resistance training facilities: [1] chest press/row, [2] hip abduction/adduction, [3] shoulder press/lateral pull-down, [4] leg press/curl, upright row/press, [5] biceps curl/triceps extension, and [6] leg extension/curl, side bend machines.

**Figure 2:**
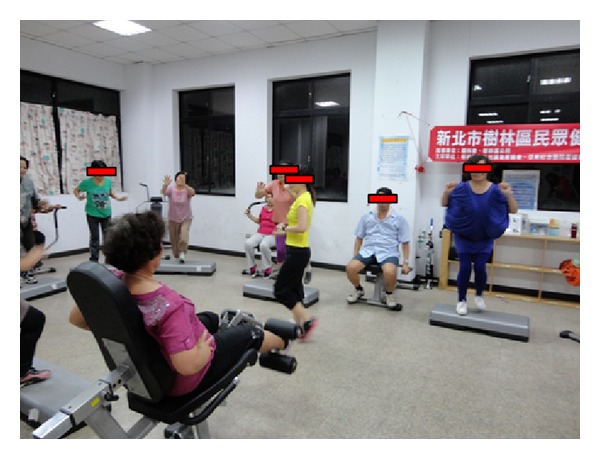
The resistance training facilities in use by the exercise intervention participants.

The exercise intervention participants participated in a 12-week exercise program (three 40 min sessions per week) in a college classroom containing hydraulic resistance training equipment. The equipment consisted of leg press/curl, shoulder press/lat pull down, hip abduction/adduction, chest press/row, upright row/press, leg extension/curl, side bend, and biceps curl/triceps extension machines. (Referring to Figure 1) Each training session began with a warm-up exercise (10 min) followed by a series of resistance training and cool-down/relaxation exercises (40 and 10 min, resp.). With music stimulation at a fixed rhythm (128–140 bpm), circuit resistance training exercises were performed within individual exercise stations interspersed with alternatively arranged 60 s stepping aerobic exercise. Each session was led by trained instructors and supervised by researchers (referring to Figure 2).

### 2.2. Center of Pressure (COP) Measurement Methods

**Figure 3:**
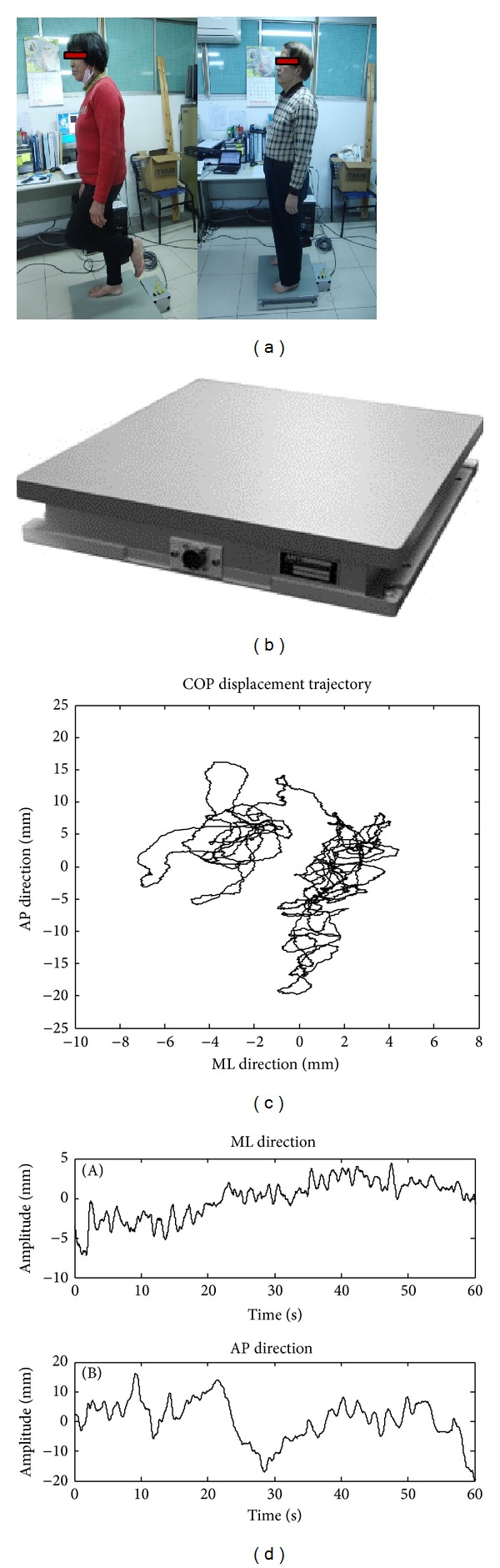
The center of pressure (COP) signal measurement and trajectory. (a) Photo of a force plate test to analyze the changes in body sway; (b) AMTI Force platform and AMTI Amplifier devices: AMTI OR6-7-2000 (Advanced Mechanical Technology, 2010); (c) displacement of COP across the force plate; and (d) measured displacement of COP in the AP and ML directions over time, respectively.

In static tests, because two-thirds of our body mass is located two-thirds of body height above the ground we are an inherently unstable system. Body movement was often measured while the subject was standing still. It was assumed that body sway reflects postural control capability in maintaining the body's center of mass within the support base. Center of pressure (COP) signals were measured along the horizontal plane in both anteroposterior (AP) and mediolateral (ML) directions. COP-related algorithms were already used as a measure of postural sway or stability [15]. Winter showed us, with the age increasing or leg muscle strength attenuation, the COP signals will produce a large amount of displacement, which means that there is a large body sway displacement [16]. In our study, Figures 3(a) and 3(b) showed a force plate testing system; Figure 3(c) illustrated the displacement of the COP in the combined AP and ML directions; and Figure 3(d) illustrated the displacement of the COP in the AP direction and ML direction, respectively.

When the displacement of the COP was large, basically represented subject's gait function was poor [17, 18]. Costa et al. used multiscale entropy analysis to measure the COP; they show that the postural sway dynamics of healthy young and healthy elderly subjects are more complex than those of elderly subjects with a history of falls [19].

### 2.3. Multivariate Empirical Mode Decomposition (MEMD)

**Figure 4:**
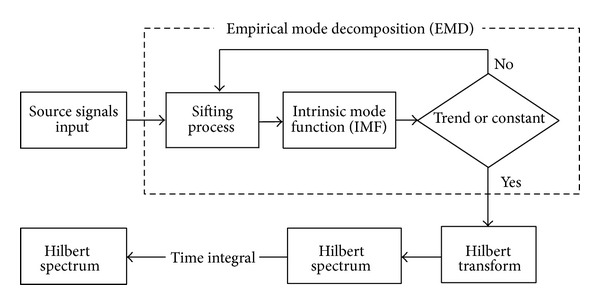
An analysis flowchart for the Hilbert-Huang transform.

The empirical mode decomposition (EMD) allows the decomposition of time-dependent time series into waveforms modulated by various amplitudes and frequencies. Since the EMD method was proposed by Huang et al. [20] it has been applied to nonlinear, nonstationary data analysis based on the intrinsic characteristics of time series. In Figure 4, we can see the analysis flowchart for the Hilbert-Huang transform. To expand the applications of EMD and MEMD, a multivariate extension of EMD was developed to decompose multivariate nonlinear and nonstationary signals [21–23].

The iterative extractions within EMD are based on local representations of the signal as a sum of the oscillating components and a residual. In other words, EMD decomposes the original signal into a set of intrinsic mode functions (IMFs), each representing embedded characteristics of the original signal. An IMF varies in both amplitude and frequency over time. In this study, the COP signal *x*(*t*) is represented as the sum of the IMFs and the final residual:

(1) $$\begin{array}{c} x\left(t\right)=\overset{n}{\underset{j=1}{\sum }}\text{IM}{\text{F}}_{j}+r_{n}, \end{array}$$

where IMF_*j*_ is the *j*th IMF, *n* is the number of extracted IMFs, and *r*_*n*_ is interpreted as the overall trend or as a residual having only one extremum in the signal *x*(*t*). For *x*(*t*), each IMF is assumed to yield a meaningful local frequency, with different IMFs exhibiting different frequencies at the same time. With the EMD procedures and sifting operations, the maximum number of sifting times (*k*) and the standard deviation (SD) of two consecutive sifting results are used to extract each IMF. Once the first IMF is obtained, the same procedure is applied iteratively to the residual *r*_*n*_(*t*) = *X*(*t*) − *d*(*k*) to extract the remaining IMFs. The standard stopping criterion terminates the sifting process only after the above condition for an IMF is met for *S* consecutive times [24].

The EMD's principles and algorithm are the same as existing multivariate extensions of EMD [22], which is a fully data-driven method for the multiscale analysis of nonlinear and nonstationary real-world signals.

The complexity index (CI), based on multiscale entropy (MSE) algorithm, has been applied in recent studies to show a person's adaptability to intrinsic and external perturbations. A higher CI value indicates a greater degree of adaptability to an external environment, while a lower CI value indicates poor adaptability [25].

The MSE method is effective in evaluating the signal complexities over different time scales and has been effectively applied in physiological, biological, and geoscientifical data analyses [10, 26]. The MMSE algorithms proposed by Ahmed and Mandic [11, 27] proposed rigorous incorporation of multivariate sample entropy to account for both within- and cross-channel dependencies in multiple data channels and then evaluate its effects over multiple temporal scales. The MMSE algorithm was shown to provide a reliable assessment of the underlying dynamic richness of multichannel observations and allows more degrees of freedom for analysis compared with standard MSE. The benefits of the proposed approach were demonstrated by simulations on complexity analysis of multivariate stochastic processes and on real-world multichannel physiological and environmental data. In the present study, the multivariate multiscale entropy (MMSE) and MSE data of the experimental group were analyzed based on the methods of Wei et al. [28], and the MMSE's principles and algorithms are similar to those in other studies. A more detailed description of the algorithm appears elsewhere [14, 27].

In this study, to acquire the COP data, subjects were rested for 3–5 min and then three continuous measurements were made interspersed by 1 min intervals of rest (referring to Figure 3). The average of the two closest measurements was used for subject analysis. Other than the COP data, results from the single-leg stance with eyes open and timed up-and-go tests were analysis using the paired* t*-test to assess training versus pretraining; posttraining versus pretraining, and detraining versus pretraining outcomes.

**Figure 5:**
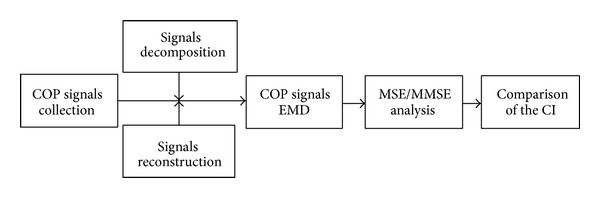
A functional module flowchart for COP signal analysis.

To apply the COP signal data for assessment of postural stability, we have included the four stages about (1) experimental COP signals collection; (2) signals decomposition and reconstruction by EMD; (3) multiscale entropy (MSE) or multivariate multiscale entropy (MMSE) analysis; and (4) comparison between traditional COP analysis and the complexity index (CI) as shown in Figure 5.

We have proposed an original multivariate multiscale methodology to assess the complexity of physiological signals. The proposed technique can incorporate simultaneous multichannel data analysis as a unique block within a multiscale framework. The basic complexity measurement is performed via the permutation entropy method to process the factor of a time series in the ordinal analysis. Permutation entropy is conceptually simple, structurally robust to noise and artifacts, and computationally rapid, which is relevant to the design of portable diagnostics.

Because time series are derived from biological systems and can be measured by multiple spatial-temporal scales, the proposed technique may present useful applications for other types of biomedical signal analysis [29].

## 3. Results

We measured gait function parameters and postural stability indicators in quadruplicate for each participant. Data from baseline (pretraining), 6 weeks (training), 12 weeks (posttraining), and 16 weeks (detraining; after training for 1 month) were collected. The 16-week data were collected to determine whether training after a period of time benefitted or worsened the patient's condition. Therefore, we requested that the study participants maintained their training regimes at home to collect postural stability indicators 1 month after completion of the supervised training programs. Of the 30 voluntary participants, only 24 completed the training regimen. The frequency statistical data of the exercise group were as follows.

Table 1 illustrates the traditional indicators of exercise group balance training are presented as “pretraining,” “training,” “posttraining,” and “detraining.” Overall, the “pretraining” scores were the poorest. As shown, the time for the “single-leg stance with eyes open” test increased from 18.10 s to 27.01 sec (*P* = 0.0651), the “timed up-and-go” test decreased from 7.67 s to 6.76 sec (*P* = 0.00013) and that of “the area of foot substrate support” increased from 392.00 ± 85.40 cm^2^ to 441.73 ± 87.72 cm^2^ and ended at 433.20 ± 90.35 cm^2^ (*P* = 0.0088) over the 12-week period. The paired* t*-test results showed statistically significant differences between the pretraining and posttraining periods after 12 weeks (*P* < 0.05). Those indicators results showed the benefit of the resistance training in the elderly.

To evaluate postural stability and intervention effects of resistance training in elderly, chronically ill patients, we employed the MSE and MMSE algorithm to calculate the relative complexity index of reconstructed COP signals in the anteroposterior (AP) and mediolateral (ML) directions via the MEMD method. The total CI values of the MSE and MMSE measurements were evaluated using the paired* t*-test.

The total MSE CI value increased and the mediolateral plane appeared to be better than the anteroposterior plane. The total MMSE CI showed the best discrimination among the other postural stability indicators. The paired* t*-test showed statistical significance between the pretraining versus posttraining (*P* = 0.0094) and pretraining versus detraining (*P* = 0.0367) sessions.

We needed a better method of analyzing the nonlinear data derived from the COP signals. Our data confirmed that the MMSE method was a better measure than MSE and other methods to serve as an indicator of postural stability.

**Figure 6:**
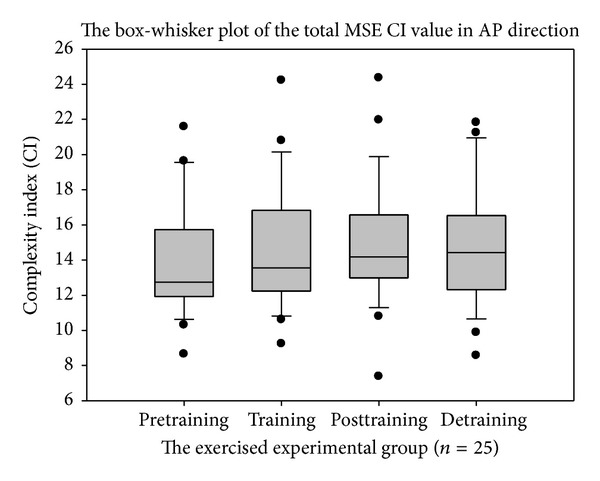
The complexity index (CI) value of total multiscale entropy (MSE) in anterioposterior plane (AP) direction.

**Figure 7:**
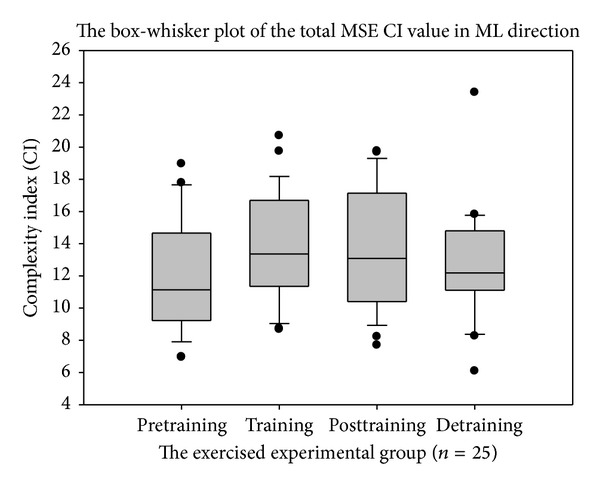
The complexity index (CI) value of total multiscale entropy (MSE) in mediolateral plane (ML) direction.

**Figure 8:**
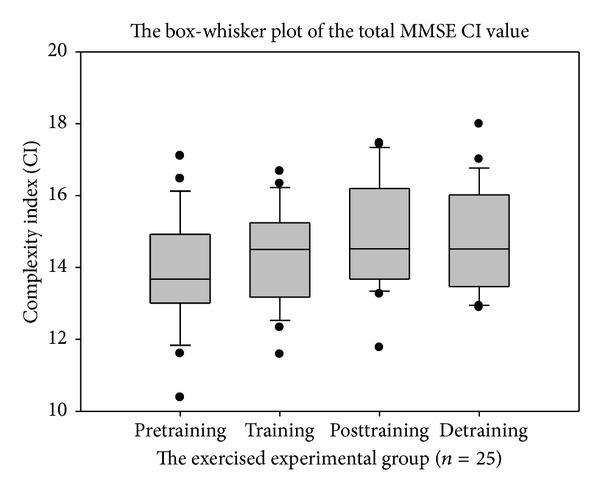
The complexity index (CI) value of total multivariate multiscale entropy (MMSE).

The box-whisker plots presented in Figures 6, 7, and 8 showed the differences in MMSE, MSE-AP, and MSE-ML between the pretraining and posttraining periods in the exercised experimental group. In Table 1, the paired* t*-test results showed statistically significant differences between the pretraining and posttraining periods after 12 weeks (*P* < 0.01) in MMSE, but there were no significant differences in MSE-AP, MSE-ML, or Balance Gait Traditional Index (TI). However, after a 12-week training program, the total CI value was enhanced in MSE-AP and MSE-ML. The mean of the “total CI of MSE in AP direction” increased from 13.88 ± 3.20 to 14.89 ± 3.47 and the mean of the “total CI of MSE in ML direction” increased from 11.95 ± 3.38 to 13.47 ± 3.74. These changes possibly resulted because the variability in MSE-AP and MSE-ML were large compared with MMSE; therefore, these differences were not statistically significant. After a comprehensive reference to Table 1, we found that compared with the MSE method as well as other sense of postural stability indicators or the traditional postural stability gait indices, the MMSE method was superior in terms of COP detection.

**Figure 9:**
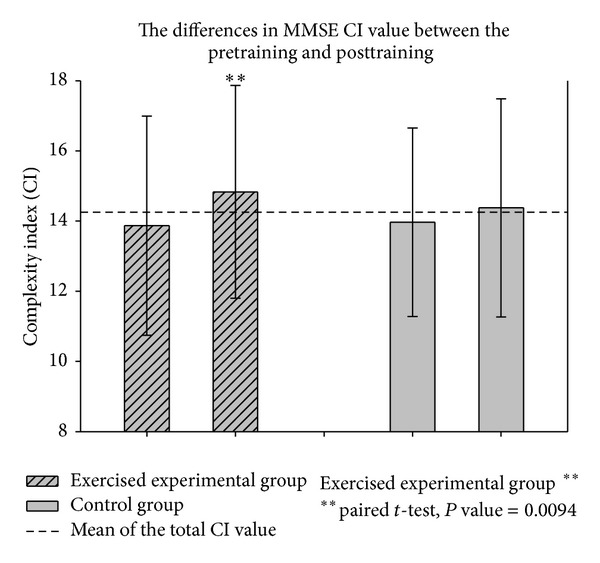
The differences in total MMSE CI value between the pretraining and posttraining.

Figure 8 illustrates the results of the MMSE method, which was used to calculate CI values of the COP data. Paired* t*-test results showed statistically significant differences in CI values between the pretraining versus posttraining periods (*P* = 0.0094) and the pretraining versus detraining periods (*P* = 0.0367) for the exercise intervention participants as shown in Figure 9 and Table 2.

To compare in greater detail the differences between pretraining and posttraining results, the frequency of statistical data in various gender and age groups for the exercise intervention participants is presented in Table 2. We further analyzed gender and age differences; participants aged approximately 45–65 years showed better results than those aged >65 years (*P* = 0.0109). Our observations were similar to those reported by Martinez-Amat et al. [9]. In this study, however, we found that females showed better results than males (pretraining versus posttraining, *P* = 0.0227; pretraining versus detraining, *P* = 0.0023). These findings were in contrast to those reported by Sherk et al. [30]. Men and women were similar in age, although men were significantly heavier and taller than the women in the study (*P* < 0.01).

## 4. Discussion

Recently published articles about MSE or MMSE algorithms reflect their contributions and benefits for applications including the analysis of pathologic heartbeat conditions such as erratic cardiac arrhythmia and congestive heart failure [31], complex dynamics of human red blood cells [32], electroencephalogram (EEG) analysis in patients [12, 13, 33], and postural sway dynamics analysis [34–36]. All the above results support the general “loss of complexity” behavior when a living system undergoes change from its normal “unconstrained” state (healthy) to that under stress due to factors such as aging and disease [37]. However, the application is still not widespread in medical clinical studies.

In the literature review [6, 9, 38, 39], to evaluate the sense of balance or postural stability, traditional clinical trials about training exercise program intervention to enhance gait function in sports medicine, rehabilitation medicine, or related fields typically use indicators of the Berg balance scale test (BBS), such as the knee flexion strength, sit-to-stand movement, timed up-and-go test, single-leg stance with eyes-open/eyes-close, and speed of walking in a straight line. All of these tools require much time to collect data, and the data may consist of high variation.

There are few studies comparing the differences between healthy young and elderly gait or balance function, using the COP data to analyze related indicators, such as the MSE algorithm generated CI of anterioposterior plane (AP) and mediolateral plane (ML), and compare them with the postural stability Gait Traditional Index (TI): for example, total distance (cm), total AP distance (cm), total ML distance (cm), average rate, average AP rate, and average ML rate. Jiang et al. recorded COP data from the healthy young and elderly subjects: (1) standing in attention, (2) standing with eyes open/closed, and (3) wearing vibrating shoes. The MSE analysis results are compared with traditional COP stabilogram metrics [10]. Using multiscale entropy analysis, the results show that the postural sway dynamics of healthy young and elderly subjects are more complex than those of elderly subjects with a history of falls [35]. However, related methods in clinical studies of patients are rare.

The most recent publications have already begun to use the MMSE algorithm to analyze the COP data as an indicator of gait function [11, 28, 36]. Ahmed and Mandic found that due to aging and the associated constraints, the complexity of postural sway for the elderly is lower than for the young [11]. Wei et al. proved that a preventative solution can be to use white noise and vibration shoes to improve the sense of balance in the elderly [28]. Bravi and Sabatini presented an original attempt to model postural sway in terms of complex-valued signals. They investigated interesting analytic properties of the bidimensional fluctuations of the center of pressure (COP) during static posturographic tests (postural sway) and compared them with the standard deviation of the AP and ML components. The results obtained in this paper clearly show that a COP model created in the complex domain is statistically different from any model developed in the real domain [36]. We seem to get similar results in that the new advanced algorithm performs better than the AP and ML methods, but we study quite different subjects. Sabatini used healthy young adults (6 males, 5 females; age: 29.6 ± 4.7, mean ± standard deviation, range: 24–38 years), but we used elderly, chronically ill patients (14 males, 11 females; age: 67.6 ± 10.7, mean ± standard deviation, range: 47–89 years).

In short, the existing literature focuses on healthy people, young or elderly, and thus did not do the benefit analysis to compare with the clinical relevant indicators in which we are interested. This study provides a comparative analysis of the results, although it uses more difficult and time-consuming measurements to get the traditional clinical indicators. However, their stability and the value of application should be better than the indicators of CI of MSE (AP, ML) and postural stability TI. In this study, we found that the CI index from the advanced MMSE algorithm seems to offer the same result; typically clinical associated indicators with high discrimination can be distinguished and significant difference between the before and after resistance exercise intervention is revealed.

This study aimed to verify the MMSE with the COP signal as a balance indicator to be applied in clinical gait analysis. The contributions of our results provided some different points in comparison with other literatures, as follows.

1. In this study, all the subjects of the patients had signed Institutional Review Board (IRB) agreements before participation. We compare the CI of MMSE with that of MSE; postural stability COP TI, and traditional clinical gait function indicators. The results showed that MMSE can be used in clinical research and proved its discrimination in this study.
2. In past studies, to evaluate the sense of balance or postural stability, traditional clinical trials about exercise training program intervention to improve gait function relied on indicators from sports medicine; for rehabilitation medicine or related fields, the MMSE should provide a reliable, yet cheaper, analysis of indicators.
3. Recent articles about MMSE-related clinical application focused on analysis of the EEG signal of critically ill patients to help distinguish coma or QBD state. A few studies compared the postural sway or gait balance function in healthy young and elderly people. However, comparison of the gait functions in elderly, chronically ill patients after exercise intervention is novel.

In the hospital, strategies for preventing falls by patients usually focus on those patients recovering from strokes and undergoing rehabilitation after surgery or hospitalized in-patients with serious illnesses but rarely focus on prevention of falls by clinic outpatients.

In this study, we used an easier and cheaper measurement tool to analysis the body sway and gait function in elderly, chronically ill patients. Explored and analyzed regular resistance exercise training can improve the balance; in addition to the elderly, people with chronic diseases are also able to enhance postural control capability and gait function. This resistance training study, conducted for outpatients over 12 weeks, not only presents the results of resistance training but also improves the potential intervention in elderly outpatients with chronic compromised gait function and sense of postural stability. Our results showed that after 12 weeks of exercise training, the subjects had significant improvement for the relevant indicators, including substrate support, timed up-and-go, and CI of MMSE (*P* < 0.05). These are similar to the results reported by Rubenstein et al., in which exercisers showed significant improvement in measures of endurance and gait; exercisers had a 10% increase (*P* < 0.05) in distance walked in six minutes and improved (*P* < 0.05) scores on an observational gait scale [38]. We found MMSE to be more sensitive and specific than other methods of measuring the COP data for postural stability and/or gait function. Meanwhile, there are more clinical studies used the MSE or MMSE method to analyze complexity or nonlinear data.

This study, in addition to providing a new and more sensitive method of measuring postural stability, also found that, for elderly home-based outpatients with chronic diseases and ailments, who require frequent visits to the hospital, together with relevant training or health education, regular resistance training may enhance their gait and postural stability functions. Such training may reduce their risk of falls in the clinic and may further reduce their risk of falling or accidental injury at home home or in the community.

## 5. Conclusions

In conclusion, circuit hydraulic resistance training programs can improve the sense of postural stability in elderly, chronically ill patients. We provide a more sensitive and specific method to measure postural stability and/or gait function. Meanwhile, there are more clinical studies used the MSE method to analyze the complexity. In this study, MMSE was more sensitive than other indicators and can distinguish differences between pretraining and posttraining sessions in the elderly. Thus, we obtained conclusions as follows.

1. According to our data, the MMSE method was superior in regards to COP detection because its discriminatory ability was superior to that of the MSE method as well as other sense of postural stability indicators or the traditional balance gait indices. And it should be a good method to deal with the nonlinear data and calculate the complexity index (CI) to assess the degree of adaptability to an external environment.

In the statistical analysis, MMSE proved more significant than MSE in dealing with the nonlinear data, such as the COP signal analysis. However, the results themselves warrant further discussion, and the methods we used need improvement. Because there was less variation using MMSE than using traditional indicators, such as single-leg stance, timed up-and-go, and others, MMSE may be a useful auxiliary indicator in the assessment of postural stability or gait function in the future.

(2) Regular exercise regimens, such as resistance training, should enhance the sense of postural stability in elderly patients, and gender and age significantly affect training outcomes in elderly, chronically ill patients.

To enhance gait function or the sense of postural stability in elderly should be a good way to avoid falls in the workplace, community, and/or home especially for patients with chronic diseases. This study results showed us that the regular resistance training exercise will improve the postural stability in elderly, chronically ill patients. In order to improve health or to avoid accidental injury, we recommend that the elderly should be exercising regularly.

This study suffered from the following two limitations.

1. The study needed volunteer participants, and at each intervention participants had to be willing to accept the resistance training exercise for 12 weeks. Further, we needed to exclude those patients unsuitable for exercise. All chronically ill patients among the participants also needed to complete the signed IRB agreements before participation. Thus, sample size was a limitation in the current study.
2. Even though the variation in the data collected from the force plate for each COP measurement was less than that for traditional indicators, such as single-leg stance, it was still large.

## Figures and Tables

**Table 1:** Patient characteristics and analytical results of the exercise intervention participants.

Participants: *N* = 25 (Mean ± SD)	Total	Pretraining	Training	Posttraining	Detraining
BMI (kg/m^2^)		25.00 ± 2.87	25.07 ± 3.08	25.17 ± 3.16	24.93 ± 3.12
Substrate support^f^ (cm^2^)		392.00 ± 85.40	441.73 ± 87.72∗	433.20 ± 90.35*^a^	439.44 ± 84.54∗
Single-leg stance with eyes open (s)		18.10 ± 22.49	33.06 ± 42.88	27.01 ± 29.00^b^	24.60 ± 24.38
Timed up-and-go test (s)		7.67 ± 2.12	7.91 ± 2.78	6.76 ± 1.97*^c^	7.28 ± 2.23
CI of MSE: anterioposterior plane (AP)		13.88 ± 3.20	14.57 ± 3.52	14.89 ± 3.47	14.67 ± 3.31
CI of MSE: mediolateral plane (ML)		11.95 ± 3.38	13.62 ± 3.31	13.47 ± 3.74	12.71 ± 3.38
postural stability Gait Traditional index (TI)					
The total distance (cm)		0.26 ± 0.18	0.36 ± 0.67	0.42 ± 0.79	0.26 ± 0.14
The total AP distance (cm)		0.20 ± 0.15	0.29 ± 0.52	0.33 ± 0.60	0.23 ± 0.13
The total ML distance (cm)		0.17 ± 0.13	0.21 ± 0.33	0.25 ± 0.38	0.11 ± 0.09
Average rate		13.99 ± 0.003	13.75 ± 0.011	14.01 ± 0.013	15.01 ± 0.002
Average AP rate		0.003 ± 0.002	0.005 ± 0.009	0.005 ± 0.010	0.004 ± 0.002
Average ML rate		0.003 ± 0.002	0.003 ± 0.005	0.004 ± 0.006	0.002 ± 0.002
CI of MMSE		13.87 ± 1.56	14.25 ± 1.33	14.83 ± 1.52*^d^	14.75 ± 1.44*^e^
Age (years old)	67.56 ± 10.70, mean ± standard deviation, range: 47–89 years				
14 males, 11 females	Male: 56%, female: 44%				

Paired *t*-test, *P value < 0.05. Training/posttraining/detraining versus pretraining.

Note: *P* value: ^a^0.0088; ^b^0.0651; ^c^0.0013; ^d^0.0094; ^e^0.0367.

Note: ^f^Substrate support is meaning bottom of the foot's trapezoidal area. After the subjects natural stand, measuring foot standing of its trapezoidal area.

**Table 2:** The total complexity index (CI) value of multivariate multiscale entropy (MMSE) within different gender and age group results for the exercise intervention participants.

exercise intervention participants: *n* = 25	Pretraining	Training	Posttraining	Detraining
Total CI of MMSE results (mean ± SD)	13.87 ± 1.56	14.25 ± 1.33	14.83 ± 1.52**^a^	14.75 ± 1.44*^b^
Median data (50%) for total MMSE CI value	13.68	14.50	14.52	14.52
Participants aged >65 years, *n* = 15 (60%)	14.14 ± 1.62	14.58 ± 1.29	14.94 ± 1.52	14.95 ± 1.62
Participants aged approximately 45–65 years, *n* = 10 (40%)	13.46 ± 1.45	13.76 ± 1.29	14.67 ± 1.57*^c^	14.41 ± 1.06
Male participants, *n* = 14 (56%)	14.35 ± 1.41	14.68 ± 1.38	15.17 ± 1.80	14.771 ± 1.53
Female participants, *n* = 11 (44%)	13.26 ± 1.60	13.70 ± 1.08	14.40 ± 0.96*^d^	14.72 ± 1.38**^e^

Paired *t*-test, *P value < 0.05; **P < 0.01. Training/posttraining/detraining versus pretraining.

Note: *P* value: ^a^0.0094; ^b^0.0367; ^c^0.0109; ^d^0.0227; ^e^0.0023.
